# A framework to assess the impact of applying formal criteria to check clinical relevance on top of statistical significance

**DOI:** 10.1186/1745-6215-14-S1-O101

**Published:** 2013-11-29

**Authors:** Werner Vach, Beryl Primrose Gladstone

**Affiliations:** 1 Clinical Epidemiology, Freiburg, Germany

## 

Recently, the topic of addressing clinical relevance on the top of statistical significance in the analysis of RCTs has been paid increasing attention. Several formal criteria to serve this purpose have been published.

In this talk we present a framework which allows to investigate the impact of applying such formal criteria, independent of the scale of the outcome variable. The only assumption made is that we have one clinical trial correctly powered to achieve a 90% power. All treatment effects are then expressed as fractions of the assumed effect.

In a first step we quantify the actual risk of having accepted a treatment with an irrelevant effect in a successful RCT, and we find this risk to be often rather small. In a second step we investigate two popular criteria based on comparing the effect estimate or the lower bound of the confidence interval with a given threshold. We demonstrate their equivalence in large samples and quantify the impact of applying them in terms of decreasing the risk of accepting a treatment with an irrelevant effect and increasing the risk of failing to accept a treatment with a relevant effect.

Putting weights on these two types of errors, we can demonstrate that the formal criteria do often more harm than good. We discuss implications of these results on adapting sample sizes to demonstrate clinical relevance and on the application of the formal criteria if several studies are summarized in a meta-analysis.

